# Lactate mediated metabolic crosstalk between cancer and immune cells and its therapeutic implications

**DOI:** 10.3389/fonc.2023.1175532

**Published:** 2023-05-10

**Authors:** Seyedeh Sahar Mortazavi Farsani, Vivek Verma

**Affiliations:** ^1^ Section of Cancer Immunotherapy and Immune Metabolism, The Hormel Institute, University of Minnesota, Austin, MN, United States; ^2^ Masonic Cancer Center, University of Minnesota, Minneapolis, MN, United States

**Keywords:** Warburg effect, cancer, lactate, glycolysis, immunotherapy, metabolism, TCA cycle, mitochondria

## Abstract

Metabolism is central to energy generation and cell signaling in all life forms. Cancer cells rely heavily on glucose metabolism wherein glucose is primarily converted to lactate even in adequate oxygen conditions, a process famously known as “the Warburg effect.” In addition to cancer cells, Warburg effect was found to be operational in other cell types, including actively proliferating immune cells. According to current dogma, pyruvate is the end product of glycolysis that is converted into lactate in normal cells, particularly under hypoxic conditions. However, several recent observations suggest that the final product of glycolysis may be lactate, which is produced irrespective of oxygen concentrations. Traditionally, glucose-derived lactate can have three fates: it can be used as a fuel in the TCA cycle or lipid synthesis; it can be converted back into pyruvate in the cytosol that feeds into the mitochondrial TCA; or, at very high concentrations, accumulated lactate in the cytosol may be released from cells that act as an oncometabolite. In immune cells as well, glucose-derived lactate seems to play a major role in metabolism and cell signaling. However, immune cells are much more sensitive to lactate concentrations, as higher lactate levels have been found to inhibit immune cell function. Thus, tumor cell-derived lactate may serve as a major player in deciding the response and resistance to immune cell-directed therapies. In the current review, we will provide a comprehensive overview of the glycolytic process in eukaryotic cells with a special focus on the fate of pyruvate and lactate in tumor and immune cells. We will also review the evidence supporting the idea that lactate, not pyruvate, is the end product of glycolysis. In addition, we will discuss the impact of glucose-lactate-mediated cross-talk between tumor and immune cells on the therapeutic outcomes after immunotherapy.

## Introduction

Animal cells, particularly the actively dividing cancer cells rely heavily on glucose as a source of energy for their survival and for generation of macromolecules required for their proliferation (1). Similarly, the fate of immune cells, their ability to get activated and their effector functions are tightly coupled with the glucose metabolism, especially during the acute phase of antigen mediated activation (2). Because cancer and immune cells rely on similar fuel types for their proliferation and activation, there is an acute competition between the two cell types for nutrients (3). Ultimately, the nutrient availability, optimal utilization of available nutrients, and presence of appropriate metabolic machinery to support the nutrient utilization decides the outcomes of cell metabolism (4). Hence, a thorough understanding of the regulators of metabolism in cancer and immune cells, especially in the context of complex environment of tumors is important for generation of appropriate immune functions and for institution of adequate anti-cancer therapies.

## Continuum of metabolism as the driver of cell functions

**Figure 1:**
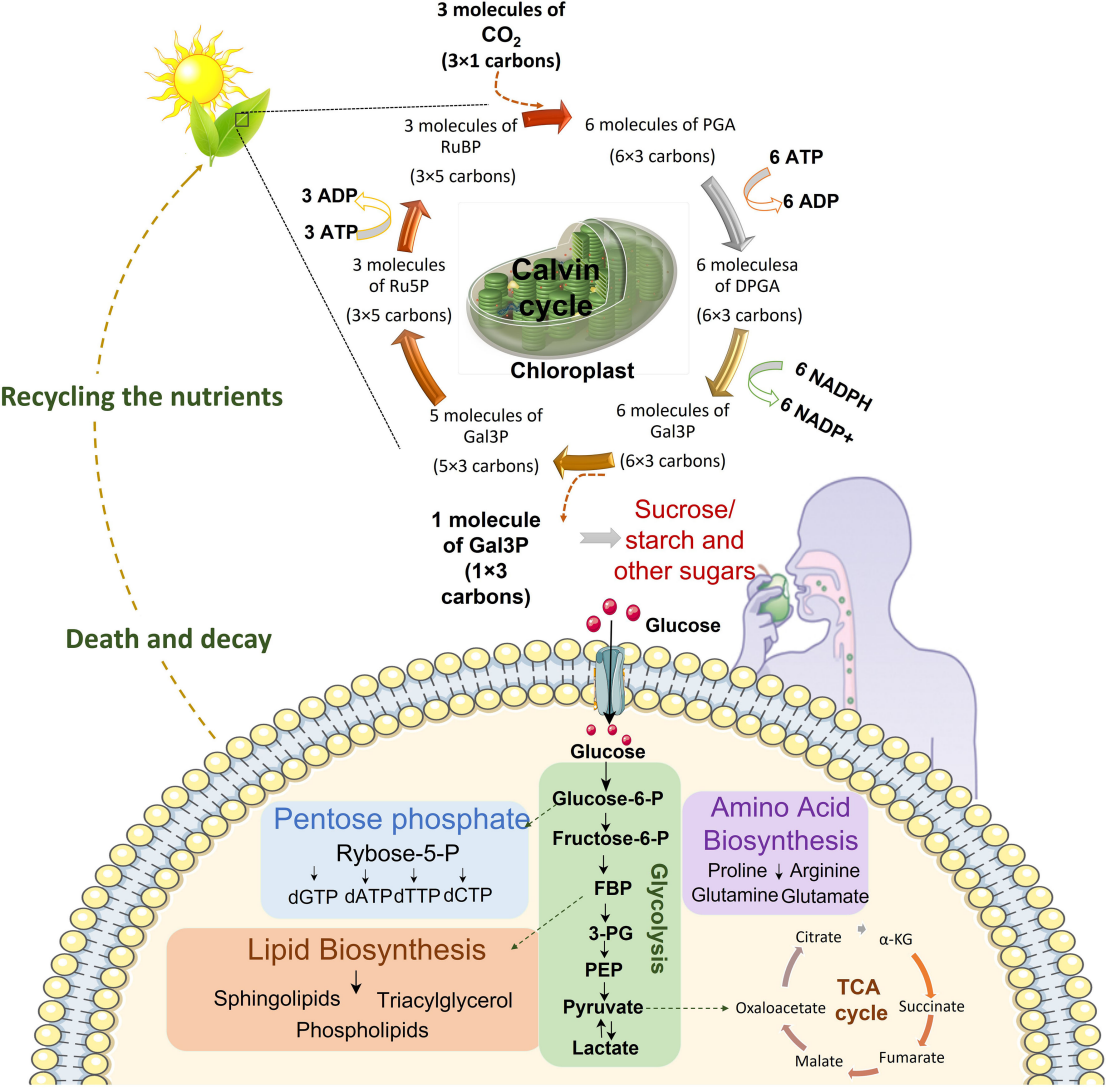
Continuum of energy flow. Central carbon metabolism is responsible to lead carbon from nutrients into biomass. The first source of carbon is atmospheric CO_2_. Plants utilize sunlight, CO_2_ and H_2_O to trigger chloroplast factory. In the chloroplast sunlight dependent reactions prepare NADPH and ATP for Calvin cycle which finally produces comestible source of carbons including sucrose, sugar and starch. These food sources would be broken down to glucose in the body. Cells uptake glucose and metabolize it in glycolysis pathway which prepare energy and various metabolites for nucleotide synthesis, lipid biosynthesis, amino acid biosynthesis and mitochondrial TCA cycle for electron reduction and energy generation. After death decay, nutrients be recycled for plants in the soil. (DPGA: Diphosphoglycerate, FBP: Fructose 1,6-bisphosphate, 3PG: 3-Phosphoglyceric acid, Gal3P: Glyceraldehyde-3-phosphate, PEP: Phosphoenolpyruvate, PGA: Phosphoglyceric acid, RuBP: Ribulose 1,5-biphosphate, Ru5P: Ribulose-5-phosphate).

The term life refers to the ability of an organism or a cell to grow, reproduce, and demonstrate functional activity and continued change preceding death (5). These life processes are supported by the sum of chemical changes termed metabolism that take place inside an organism at cell and molecular levels, leading to generation of energy or building blocks required for sustenance of life (6). Metabolism not only provides the energy and building blocks for cellular growth but also ensures protection against stress factors such as osmotic changes, xenobiotics, and oxidative stress (7). Metabolism has evolved to support cell function and activity by either generating or breaking down the building blocks, based on which the metabolism can be respectively termed anabolic or catabolic. In anabolic metabolism, utilizing simpler building blocks such as glucose, free fatty acids and amino acids, cells synthesize complex molecules such as glycogen, fatty acids, and proteins which are required for generation of cellular building blocks (8). On the contrary, catabolism refers to the breakdown of complex cellular molecules into their simpler forms. Hence, anabolism and catabolism represent two opposite ends of the metabolic spectrum (9). In particular, the central carbon metabolism that represents the six carbon fixation pathways, ensures conversion of carbon and energy sources such as sugars into precursor of metabolism which are used to generate entire biomass of the cells in addition to the generation of free energy, redox power, and precursor metabolites required for biosynthesis (**Figure 1**). Depending upon the cellular/organismal complexity, the amount of cell’s genetic and proteomic machinery involved in regulating metabolism varies. Metabolism is usually the largest constituent of the proteome with approximately 50% of the proteome being allocated to metabolism in yeast. In humans the fraction of proteome associated with cell metabolism is lower as a larger fraction of the proteome is allocated to cell signaling, cytoskeleton proteins, chaperones, and the spliceosome (10). However, consistently within the metabolic spectrum, the glycolytic enzymes are allocated a larger fraction of the proteome than the TCA cycle (10) with about 15-20% being allocated alone to glycolysis in humans (11). The high catalytic efficiency, small size, and high abundance of enzymes in the central carbon metabolism are consistent with the central role this part of metabolism plays in ensuring constant provision of energy, primarily in the form of ATP, in handling electron flows by balancing the co-factors NADH and NADPH, and in providing precursors for cellular growth (12). Thus, the flux through the central carbon metabolism typically exceeds the flux through other metabolic pathways by a factor of 10 or more. With these multiple roles, the central carbon metabolism must be highly connected with the other parts of metabolism (12). This implies that a perturbation of almost any part of metabolism results in a global response in which a large number of enzymes have to alter their function in order to maintain homeostasis or generate a particular cell function such as effector functions in T cells (13). This explains why almost any change in cellular physiology has a metabolic fingerprint, i.e., changes in a certain part of metabolism. Thus, it is safe to say that metabolic perturbations have a global impact on cell function and physiology (13). In this review, we critically analyze the intricately associated central carbon metabolism in cancer and immune cells with special reference to glycolysis and lactate metabolism. We provide evidence that glucose derived lactate may be a significant driver of mitochondrial metabolism in CD8 T cells and that lactate driven cross talk between tumor and immune cells shapes the response to therapies. We also discuss the potential of targeting central carbon metabolism as an avenue for enhancement of anti-cancer therapies.

## Regulation of glycolysis

Central carbon metabolism plays an important role in metabolic networking and is composed of the flow of carbon from nutrients into biomass. Central carbon metabolism is composed of the glycolytic pathway, the citric acid cycle, the pentose phosphate pathway (PPP), and six known carbon fixation pathways (14). Of these, carbon fixation pathways are the most fundamental pathways that take place inside the mesophyll cells of plants that help to bring CO_2_ into the anabolic phase of cell metabolism (15). Sugars, primarily glucose, fuels the glycolytic pathway in animal cells whereby through a series of enzymatic reactions these sugars are broken down into pyruvate which is then either fed into the mitochondrial TCA cycle for electron reduction and ATP generation or is converted into lactate in the cytoplasm. The PPP shunts carbons back into the glycolytic or gluconeogenic pathways and is a major regulator of the cellular reduction-oxidation (redox), homeostasis and biosynthesis. Glycolysis and citric acid cycle (also called tricarboxylic acid (TCA) cycle) are the most intricately associated and well-defined energy generating pathways in eukaryotic cells. In glycolysis glucose, through a multistep reaction is converted into pyruvate which is then transformed either into lactate that is secreted to outside of the cell or gets converted into oxaloacetate (OAA) or acetyl-CoA that feeds into TCA cycle inside the cell (**Figure 1**). In the presence of adequate amounts of oxygen, cytoplasmic glycolysis is connected to the mitochondrial respiratory chain that enables oxidative phosphorylation (OXPHOS) by transport of electrons through the proteins of the respiratory chain (16). This electron transport generates a proton gradient which is necessary for ATP synthesis. Ideally, when glycolysis and OXPHOS are coupled, one mole of glucose produces up to 36 moles of ATP. However, under the conditions of limited oxygen availability, OXPHOS reactions are impaired, and there is a compensatory upregulation in the glycolytic activity that helps to fulfill the increased energy demands (17). Even if the oxygen concentrations are high, if the demand for ATP increases suddenly, such as under acute cell expansion phase after antigenic stimulation of immune cells, aerobic glycolysis is enhanced rapidly since mitochondrial activity is not sufficient to supply the required amount of ATP. Moreover, intermediate metabolites of glycolysis are precursors for the biosynthesis of pentose phosphates, hexosamines, glycerophospholipids and amino acids, so that glycolysis can fuel various anabolic pathways whenever required. Hence, an upregulated glycolytic pathway not only supplies ATP under acute energy shortage conditions, but also provides intermediates for cell biomass synthesis.

Glycolysis is regulated at three points, each serving a different function. Hexokinase (HK), phosphofructokinase (PFK) and pyruvate kinase (PK) are the three rate-limiting enzymes regulating the glycolytic flux. HK controls the entry of glucose into the glycolytic pathway by producing glucose-6-phosphate (G6P), which also acts as an allosteric inhibitor of HK. HK exists in 4 isoenzyme types (HK1-4) with HK1 and HK3 being ubiquitously expressed while HK4 being restricted to liver and pancreas. HK1-3 are associated with the outer mitochondrial membrane and are shown to play a critical role in maintaining aerobic glycolysis in cancer cells. High affinity HK2 is mainly expressed in tissues with high energy demand such as tumors. In particular, HK2 has been shown to act as a bridge between cell metabolism and cellular longevity primarily by preventing the mitochondrial death pathways (18). Surprisingly, HK2 has been found to be dispensable for T cell based immunity (19) thus pitching HK2 as a putative differential target in tumor cells that heavily rely on HK2 for their energy and biosynthetic demands (20). High expression of HK2 in tumor and associated mesenchymal stromal cells inhibit glucose uptake in T cells preventing their activation. The second point of glycolysis regulation is the entry point of fructose-6-phosphate into glycolytic cycle by phosphofructokinase (PFK). PFK exists as a tetramer and has two isoforms, PFK1 and PFK2. PFK1 catalyzes the conversion of fructose-6-phosphate to fructose-1,6-bisphosphate while PFK2 catalyzes the conversion of fructose-6-phosphate to fructose-2,6-bisphosphate. Fructose-2,6-bisphosphate is a stimulator of PFK1 by its ability to increase the affinity of PFK1 for fructose-6-phosphate and to decrease the ability of ATP to inhibit the reaction (21). When the rate of PFK1 is slowed, G6P accumulates and is routed toward glycogen synthesis or the pentose phosphate pathway (PPP). PFK-1 is allosterically regulated by effectors such as fructose-2,6-biphosphate (FBP) or adenosine monophosphate (AMP). Oncogene activation including Ras and Src leads to reduced regulation of PFK1 activity by elevated levels of FBP that acts as a natural activator of PFK1 (22, 23) leading to enhanced glucose uptake and its conversion into downstream substrates, preferably lactate that can be shunted into various biosynthetic pathways. There is limited evidence regarding the role of PFK in immune cells. In CD4 T-helper cells from rheumatoid arthritis patients, deficiency of PFK was found to impair the ATP generation and autophagy, making the cells prone to apoptosis and senescence (24). In addition, PFK seems to have a significant role in regulatory T cells, as calcium regulated protein kinase-4 (CaMK4) controlled PFK-platelet type (PFKP) was found to enhance the regulatory role of these cells (25). Finally, in an irreversible reaction pyruvate kinase (PK) controls the conversion of phosphoenol pyruvate (PEP) into pyruvate or to gluconeogenesis. Pyruvate kinase exists in four isozyme forms: PKL (liver), PKR (red blood cells), PKM1 (muscle and brain) and PKM2 (early fetal tissue and actively growing cells such as tumor cells and immune cells). PKM2 can exist in two isozyme forms, the tetrameric and the dimeric form, both of which are constituted of the same monomeric units. Tetrameric PKM2 (tet-PKM2) localizes in the cytoplasm and is the enzymatically active form while the dimeric PKM2 (di-PKM2) localizes in the nucleus and is transcriptionally active form. There are several allosteric stimulators that induce tetrameric form including F1,6-BP that help to prevent a metabolic roadblock when upstream PFK is active. In fasting conditions, pyruvate kinase is allosterically inhibited by ATP and alanine (mostly mobilized from muscle) decreasing the concentrations of tet-PKM2 that prevents PEP that is needed for gluconeogenesis from being converted directly back to pyruvate. The role of PKM2 in immune cells is not well defined and only recently has started to be appreciated. Angiari et al. show that the tetramerization of PKM2 prevents CD4 T cell activation. Most effect was on generation of Tregs and Th17 cells thus preventing the induction of autoimmune diseases (26). Similarly, PKM2 in macrophages has been shown to prevent generation of proinflammatory phenotype thus helping in prevention of autoimmune disorders (27). However, the role of PKM2 in cytotoxic CD8 T cells is still under debate.

**Figure 2:**
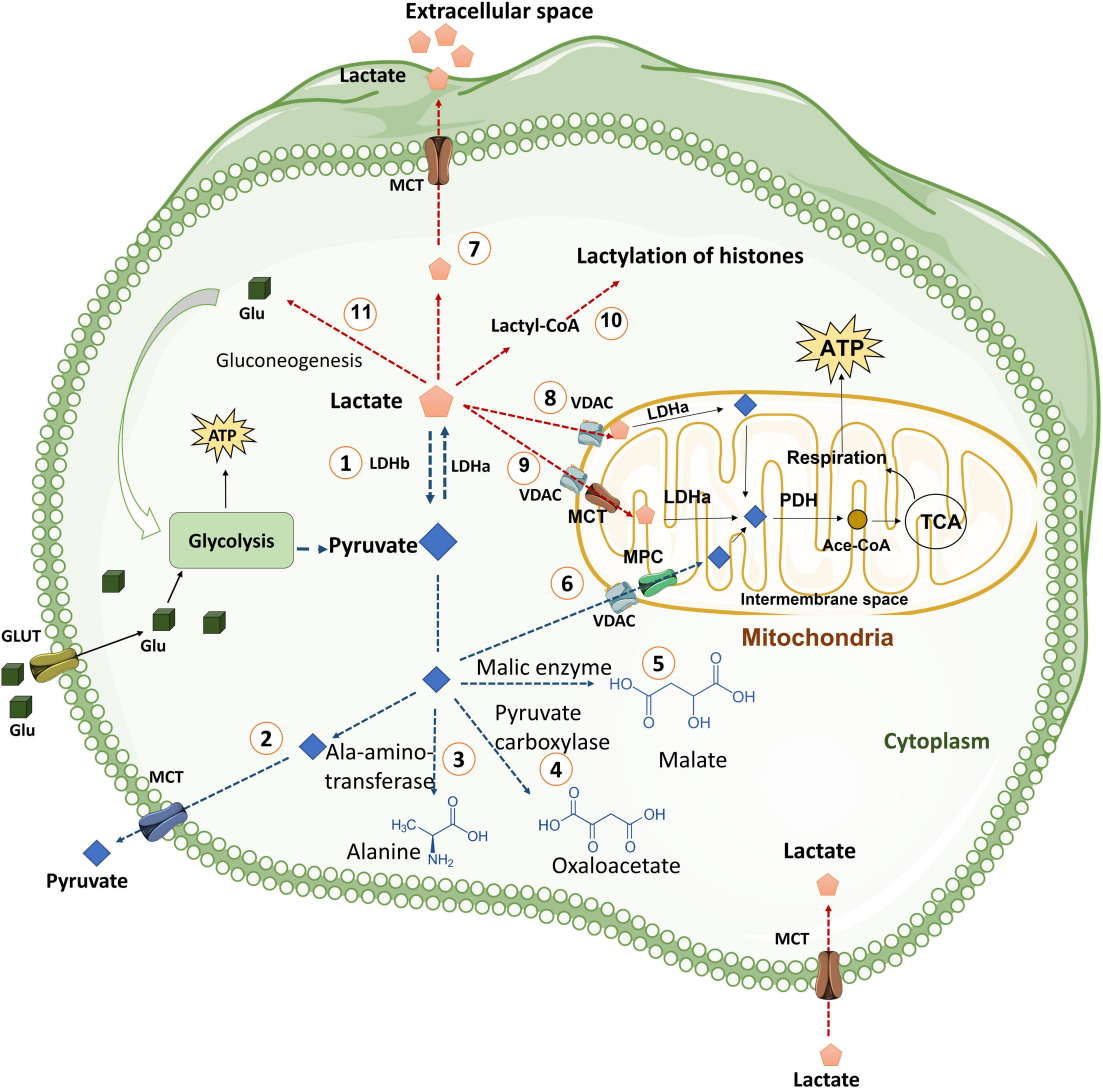
The fate of pyruvate and Lactate in the cells. Cells uptake glucose molecules *via* GLT and use them in glycolysis pathway. Glycolysis prepares ATP and some other intermediate metabolites for cells. Pyruvate derived from glycolysis has several fates including: (1) Conversion pyruvate to lactate *via* LDHa enzyme quickly. This reaction is reversible by LDHb. (2) Exporting pyruvate from cells by MCT transporter, which is located in plasma membrane, to extracellular space. (3) Generation of alanine amino acid from pyruvate *via* Ala-amino-transferase enzyme. (4) Conversion of pyruvate to oxaloacetate by pyruvate carboxylase enzyme. (5) Generation of Malate from pyruvate in a reaction mediated by malic enzyme. (6) Transporting of pyruvate into mitochondria by VDAC in the mitochondrial outer membrane and MPC transporter in the mitochondrial inner membrane. Pyruvate in mitochondria is converted to Ace.CoA by PDH and used in TCA cycle. TCA cycle generates NADH and FADH_2_ for mitochondria respiration process in the inner membrane of mitochondria that produces more ATP for cells. Lactate derived from glycolysis has also various fates including: (7) Releasing lactate from cells into extracellular space by MCT transporters (MCT1 and MCT4). (8) Transporting lactate into Intermembrane space, by VDAC channel, where lactate can be converted to pyruvate by LDHa. (9) Entering lactate into the mitochondrial matrix where lactate can be converted to pyruvate by LDHa. (10) Lactate can be converted into lactyl-CoA and is involved in the lactylation of histones in the nucleus. (11) Lactate is converted to glucose through gluconeogenesis and glucose goes back to glycolysis. (Ace. CoA: Acetyl coenzyme A, GLT: Glucose transporter, LDH: Lactate dehydrogenase, MCT: Monocarboxylate transporter, MPC: Mitochondrial pyruvate carrier, PDH: Pyruvate dehydrogenase, PEP: phosphoenolpyruvate, PEPS: phosphoenolpyruvate synthetase, TCA cycle: Tricarboxylic acid cycle, VDAC: Voltage-dependent anion channel).

Pyruvate generated as a result of glycolysis can have multiple fates in the cytoplasm. Pyruvate can be effluxed from the cell, or is converted into alanine by alanine aminotransferase, or by the process of gluconeogenesis reactions is converted into oxaloacetate or malate, or may be transported into the mitochondria where it is converted to acetyl-CoA for its utilization in the TCA cycle. Interestingly, none of these steps occur at a rate that can match the conversion of pyruvate into lactate, making lactate the inevitable and ultimate metabolite of glycolytic pathway (**Figure 2**). Pyruvate is the precursor of lactate and under certain conditions can exclusively be the source of energy inside the cells. Pyruvate is transported into mitochondria by MPC1 and MPC2 heterodimers (28, 29). In the inter-mitochondrial membrane, it gets converted into acetyl-CoA that is funneled into TCA cycle. Pyruvate alters epigenome in CD4 T cells during activation by altering the cell genome (30). In the same line, inhibition of mitochondrial transfer of pyruvate by blocking MPC1 and MPC2 has been shown to mold the CD8 T cells into memory phenotype thus supporting the observation that enhanced availability of pyruvate and its oxidation through mitochondria supports effector functions (31). Moreover, pyruvate metabolism may support antitumor signaling in CD8 T Cells by upregulating succinate uptake through its receptor (32).

Pyruvate is converted into lactate by lactate-dehydrogenase (LDH). LDH is a tetrameric enzyme composed of two protein subunits. The tetramer can be assembled by combination of the M (muscle) form (encoded from Ldh-A gene) or the H (heart) form (product of the Ldh-B gene) producing five separate isozymes: M_4_ (LDH5), M_3_H_1_ (LDH_4_), M_2_H_2_ (LDH3), M_1_H_3_ (LDH2), and H_4_ (LDH1) (33). These isozymes have different kinetic properties with respect to substrate affinity and inhibition among these isozymes. LDH activity depends on the metabolic switch to anaerobic respiration. LDH is modulated by three types of regulations, namely, allosteric modulation (34), substrate-level regulation (35), and transcriptional regulation (36). The relative availability and concentration of substrates regulate the activity of LDH. The enzyme becomes more active during high availability of its substrates. The demand for ATP compared to aerobic ATP supply causes the accumulation of ADP, AMP, and free phosphates (Pi). Glycolytic flux leads to the production of pyruvate that exceeds the metabolic capacity of pyruvate dehydrogenase and other shuttle enzymes that metabolize pyruvate. This process channelizes the flux of pyruvate and NAD+ through LDH, subsequently generating lactate and NADH (37).

## The anecdote of Warburg effect

The ability of pyruvate to get converted into lactate even under aerobic conditions has been established as a universal phenomenon. Importantly, aerobic glycolysis was traditionally considered to be a negative cellular phenomenon that contributed to cell exhaustion partly by nutrient depletion and partly by accumulation of acidic byproduct such as lactate (38). Pyruvate can be converted into lactate quickly by lactate dehydrogenase (LDH) and lactate is the final product of glycolysis that was thought to be produced as a waste material by the tumor or the tumor associated stromal cells (39). However, over a period of time several published reports demonstrate that lactate can serve as a significant source of energy inside cells. In fact, in CD8 cells, lactate has been shown to be the preferred substrate albeit in a narrow range of concentrations. Importantly, tumor infiltrating cytotoxic CD8 T cells have been shown to be dependent on lactate metabolism to sustain their antitumor function (40). It has bene shown that mitochondria are capable of transporting lactate across the inner membrane and oxidizing it (41). Lactate transport into the mitochondrial matrix would simultaneously deliver both pyruvate and cytosolic reducing equivalents from the cytosol into the mitochondrial matrix (42). There are some other evidences suggesting that LDH and monocarboxylate transporter (MCT)1 are colocalized in the inner mitochondrial membrane facilitating the transport of lactate into the mitochondria (43). Excessive lactate production and rapid lactate transport in cancer cells depend primarily on the upregulation of hypoxia-inducible factor-1α (HIF-1α) and c-Myc (44, 45). Continuous activation of HIF-1α and c-Myc causes aberrant expression of multiple glycolytic enzymes and monocarboxylate transporters (MCTs), including lactate dehydrogenase A (LDHA), MCT1, and MCT4 (46). Lactate in the TME not only induces lactic acidosis, but also shuttles among cell populations, including cancer cells, tumor-associated stromal cells, tumor-associated macrophages (TAMs), and tumor-infiltrating lymphocytes (TILs) (47, 48). Cancer cells export lactate to the extracellular space *via* MCTs (49) that makes many unpleasant consequences in tumor microenvironment (TME) (38). High level of lactate decreases pH in TME which triggers increase of angiogenesis, proteolytic activity, metastatic, and resistance to anti-cancer therapies (50). High lactate in TME also makes cancer prognosis more difficult (48). Recently, in breast cancer cells lactate has been shown to regulate malignancy by reprogramming energy metabolism and by altering cell signaling *via* binding of lactate to G-protein-coupled receptor 81 (GPR81) (51). In addition to its effect in cancer cells, an indirect effect of interaction between lactate and GPR81 is to reduce the expression of MHCII on APCs in the TME that tend to mitigate the generation of immune response and promote immune escape (52). The use of lactate or alternate molecules such as glutamine as an energy source may not only depend upon the activation of various signaling pathways but also on the anatomical location of tumor cells. For example, tumor cells located deep in the TME away from blood supply may use glutamine as a source of energy for glycolysis and produce huge amount of lactate, whereas cancer cells near blood vessels (in normoxic condition), such as in lung tumor, prefer to oxidize lactate and obtain energy by TCA cycle (53). The presence of various physiologic carbon sources (PCSs) such as lactate, acetate, glutamate, citrate, and pyruvate in extracellular environment strongly impact the uptake and utilization of glucose by CD8 T cells. Enough amount of PCSs in the cell culture media decreases glucose contribution to the TCA cycle and interestingly, enhances effector function, such as production of IFN-γ (40). The role of glutamine mediated cell metabolism is intriguing in this regard. Glutamine participates in TCA cycle and in the synthesis of nucleotides, glutathione, and other non-essential amino acids. In fact, despite being a non-essential amino acid, it is considered essential for tumor cell metabolism as its deprivation suppresses tumor cell growth and induces cell killing. Interestingly, glutamine supports mitochondrial metabolism when glucose derived pyruvate is converted into lactate. However, in the current review, we are going to limit our discussion to the effect of lactate metabolism in immune cells and its impact on immune mediated anti-cancer responses.

## Effect of lactate in different immune cell populations

Glycolytic pathway, through the active or passive participation of its metabolites controls the function of various immune cells. Active participation refers to direct effect of various metabolites on the cell function and physiology while passive control is by relative abundance, or lack of thereof, of various metabolites, particularly the glucose and lactate which may be required for energy generation or cell signaling. In this section we discuss the implications of metabolite alterations, especially increased lactate concentrations on various immune cell populations.

### Lymphocytes

T cells in the TME have been found to lose their antitumor activity because of either glucose deprivation (54), or presence of high levels of lactate. The presence of high levels of lactate (which is mostly tumor derived) disrupts the transmembrane concentration gradients thus preventing the secretion of intracellular lactate by activated and proliferating immune cells. This leads to decreased intracellular pH and shutting down of the homeostasis cell machinery tipping the balance towards cell dysfunctionality. Accumulation of lactate in T cells is mediated by the high expression of MCT1 lactate transporters after TCR engagement that increases lactate uptake into the cells (48). In the tumor-immune microenvironment, the effect of lactate on immune cells can be highly complex and hard to decipher, which is further confounded by acidic protons, a co-product of glycolysis. In one study, Mendler et al. showed that lactate acidosis impaired the TCR-triggered induction of p38/JNK signaling required for IFNγ production but not the MEK1/ERK signaling required for granule movement (55). In another study, lactate has been shown to reduce pyruvate carboxylase mediated replenishment of TCA cycle intermediates leading to inhibition of anaplerotic pathways (32). In yet another study, inhibition of lactate dehydrogenase, in combination with IL-21 was found to reduce the lactate concentrations while increasing the stemness and anti-tumor ability of CD8 T cells (56). In contrast to these studies, lactate has been shown to increase the stemness of CD8 T cells leading to augmentation of anti-tumor immunity (57). In this study, using mouse models of colon cancer, subcutaneous administration of lactate but not glucose was found to inhibit tumor growth in a CD8 T cell-dependent manner. This reduction in tumor growth was associated with an increased proportion of TFC1^+^CD8 T cells, as revealed by single cell transcriptomics analysis (57). Mechanistically, lactate inhibits histone deacetylase activity, which results in an increased acetylation at H3K27 of the *Tcf7* super enhancer locus, leading to increased *Tcf7* gene expression. In addition, *in vitro*, lactate pre-treated CD8^+^ T cells were also found to efficiently inhibit tumor growth upon adoptive transfer to tumor-bearing mice. However, one limitation of in-vitro studies is the exposure of immune cells to super-physiological conditions where nutrients are available in excess compared to physiological conditions (58) that may alter the outcomes of cell activation. Indeed, CD8 T cells activated under *in vivo* conditions were found to utilize glucose though oxidative metabolism with flow of glucose derived carbon into anabolic phase compared to *in vitro* activation that showed hallmarks of aerobic glycolysis (59). Hence, the observations from various studies should be analyzed with caution as metabolite utilization and cell activation events may be highly context dependent.

### Regulatory T cells

**Figure 3:**
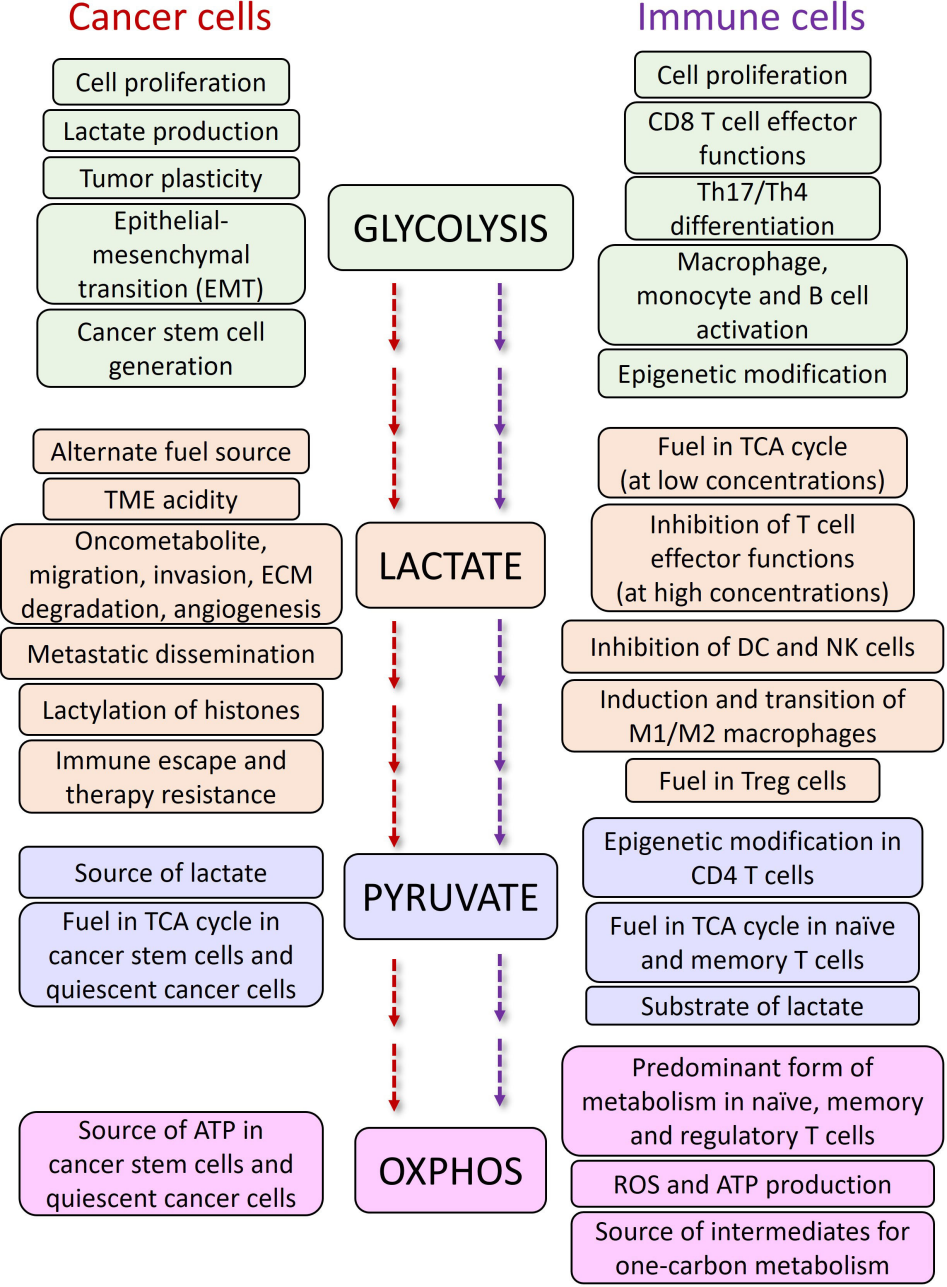
The functions of glycolysis and OXPHOS pathways and, lactate and pyruvate metabolites in cancer and immune cells.

The effects of lactate on Treg cells in TME are also in favor of cancer progression. It has been shown that lactate can preserve Treg cell immune-suppressive functions by upregulation of FOXP3 (60), and MCT1 (61). In a recent study, Gu et al. showed that tumor derived lactate regulates Tregs by lactylation of MOESIN at Lys72 residue enhancing the TGBβ mediated signaling *via* TGFβ-RI (62). The more the expression of FOXP3, the more OXPHOS, NAD^+^ oxidation and adaptation of Treg cells to low-glucose and high-lactate conditions (60). Additionally, MCT1 mediated lactate influx and intracellular lactate metabolism are important for tumor-infiltrating Treg cells to sustain their suppressive activity (63), while high glucose levels dampen their function and stability (64). A summary of the effects of glycolysis, lactate and OXPHOS on cancer and immune cells are described in **Figure 3**.

### Natural killer cells

In addition to T cells, NK cell activity is also directly and indirectly affected by high level of lactate (65). Lactate acidosis restricts the cytolytic functions of natural killer (NK) cells by inhibition of nuclear factor of activated T cells (NFAT), reducing IFNγ production and downregulation of peroxisome proliferator-activated receptor g (PPARg) (66).Tumor derived lactate has also been shown to inhibit cytotoxic NK cell activity by inhibiting the production of perforin and granzyme or indirectly by enhancing the numbers of myeloid derived suppressor cells that suppress the functionality of NK cells (67). Interestingly, tissue resident NK cells from liver have been found to have increased sensitivity to lactate that impairs the mitochondrial functions leading to cell apoptosis (65). Hence, lactate in NK cells seems to have a multipronged strategy all culminating in suppression of NK cell activity.

### Monocytes, dendritic cells and macrophages

There are two different reports about the effect of lactic acidosis on monocytes: Lactate induces monocyte differentiation to immunosuppressive dendritic cells or macrophages (68, 69), but huge amount of lactate may also delays the differentiation of monocytes into dendritic cells (70). Lactate also indirectly can be sensed by G-protein-coupled receptor 81 (GPR81, also termed hydroxycarboxylic acid receptor 1 or HCAR1) on the surface of plasmacytoid dendritic cells (pDCs). This interaction triggers calcineurin phosphatase signaling, leading to enhancement of free cytosolic Ca^2+^ and deduction of pDC function (71). Additionally, MCT1-mediated lactate influx partially contributes to the inhibition of pDC activation (72). Tumor derived lactate also increases M2 macrophage polarization mediated by ERK-STAT3 signaling pathway (73), HIF-1a stabilization (74), and G-protein-coupled receptor 132 (GPR132) activity (75). Lactate binding to the surface GPR132 in macrophages leads to induction of cyclic AMP (cAMP) and cAMP early repressor (ICER), thus increasing the expression of arginine-metabolizing enzyme arginase 1 (ARG1), VEGF (76), and HIF-1a (77), and the production of pro-angiogenic phenotype of macrophages (78). Lactate acidosis also reduces the function of M1 macrophages by downregulation of IL-6, iNOS, and CCL2 (79). Epigenetic modification mediated by high levels of intracellular lactate are also demonstrated in macrophages. Lactate inhibits NAD^+^-independent histone deacetylase and enhances histone lysine residue lactylation in macrophages (80). Histone lactylation level has been shown to have a direct correlation with oncogenic factors generation in M2 macrophages (81). In addition to its signaling effects, lactate acts as a direct Carbon source in tumor associated macrophages (TAMs), directly derogating the MHCII^hi^ TAM subset thus stimulating the T cell suppression by transcriptionally stabilizing the MHCII^lo^ TAM subset (82).

## Targeting glycolysis in solid tumors for therapy enhancement

**Figure 4:**
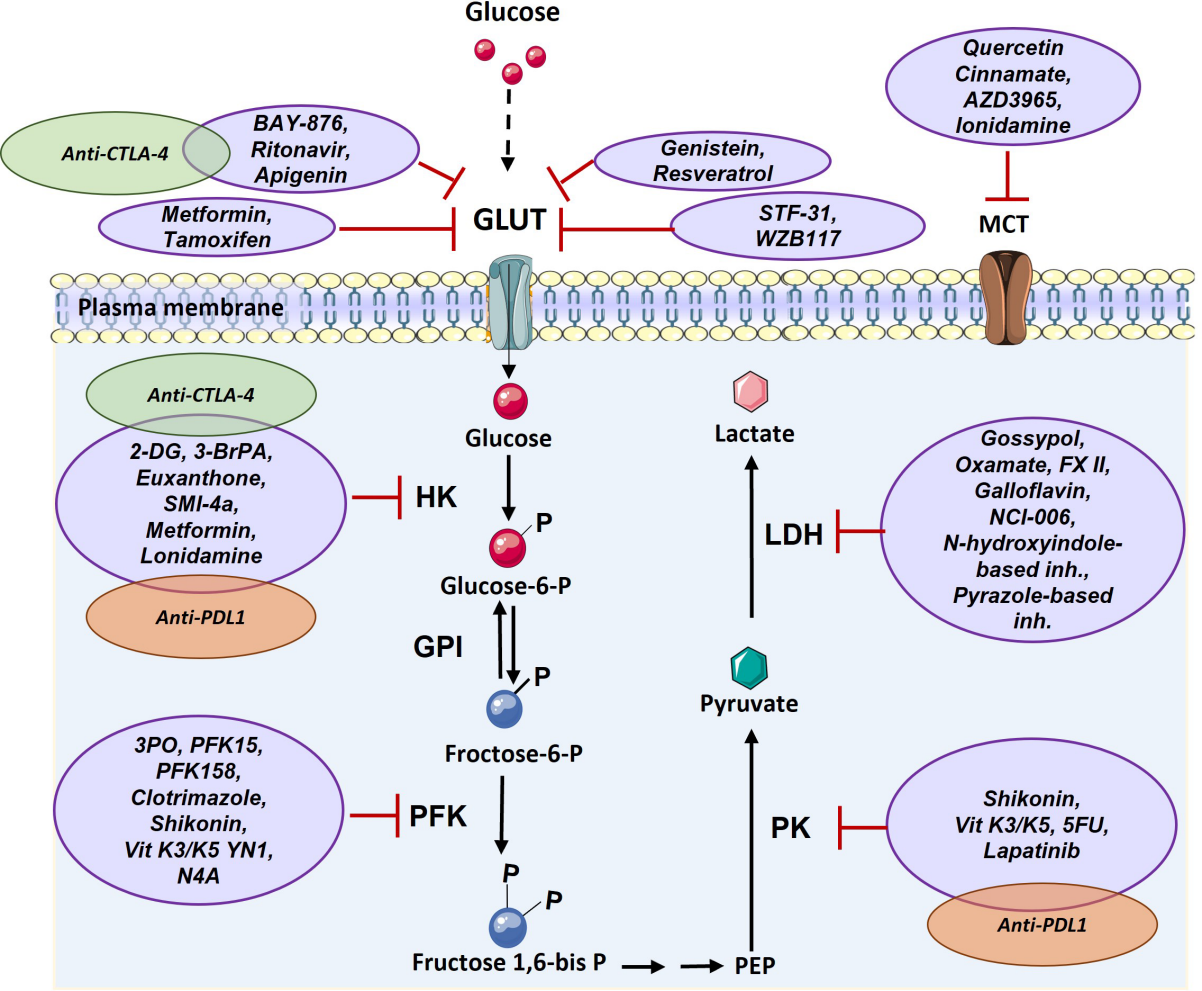
Targeting glucose transporter, critical glycolytic enzymes, and lactate transporter in cancer therapy. GLUT can be targeted *via* various components such as Ritonavir, Apigenin, Metformin, Tamoxifen, Genistein, Resveratrol, STF-31, and WZB117. HK enzyme can be inhibited by 2-DG, 3-BrPA, Euxanthone, SMI-4a, Metformin, Lonidamine. PFK enzyme is targeted by 3PO, PFK15, PFK158, Clotrimazole, Shikonin, Vit K3/K5 YN1, and N4A. PK enzyme can be inhibited by Shikonin, Vit K3/K5, 5FU, and Lapatinib. LDH enzyme is targeted by Gossypol, Oxamate, FX II, Galloflavin, NCI-006, N-hydroxyindole-based inhibitors, Pyrazole-based inhibitors and MCT transporter can be inhibited by Quercetin, Cinnamate, AZD3965, and Ionidamine. There are a few combination therapy with metabolism targeting and immunotherapy that are shown by green (anti-CTLA-4) and orange (anti-PD1) (2-DG: 2-Deoxy- d-glucose, 3-BrPA: 3-bromopyruvate, 5FU: Fluorouracil, GLUT: Glucose Transporter, GPI: Glucose-6-phosphate isomerase, HK: Hexokinase, LDH: Lactate dehydrogenase, MCT: Monocarboxylate Transporter, PEP: Phosphoenolpyruvate, PFK: phosphofructokinase, PK: Pyruvate kinase, Vit K: Vitamin K).

Given the central role of glucose mediated metabolism in cancer and immune cells (3), to use glycolysis inhibitors to impede the growth and spread of cancer cells, which could potentially help to improve the efficacy of cancer immunotherapy treatments are being developed (83, 84). Both, the process of glucose utilization and the production of lactate through glycolysis are high in cancer cells, resulting in higher turnaround of the proteins, enzymes, and metabolites passing through the pathway, thus making these proteins lucrative targets for the diagnosis and treatment of various cancers (85). There are various drugs which target glucose transferase1 (GLUT1) such as BAY-876, ritonavir, genistein, STF-31 and WZB117. These drugs inhibit glucose uptake into cancer cells and lead to cell death. After uptake of glucose, it is phosphorylated in a rate limiting reaction by hexokinase (HK), making HK another target for cancer therapy. Several drugs have been developed for inhibition of this enzyme, such as 2-deoxy-D-glucose (2-DG) and 3-BrPA. Another glycolytic enzyme with potential for targeting in cancer treatment is PFK which creates fructose-1,6-bisphosphate from substrate fructose-6-phosphate. The main PFK inhibitors include 3-(3-pyridinyl)-1-(4-pyridinyl)-2-propen-1-one (3PO), 1-(4-pyridinyl)-3-(2-quinolinyl)-2-propen-1-one (PFK15), PFK158, YN1, and N4A. Since lactate has important effects in favor of tumor growth including acidification of tumor microenvironment and triggering immune suppressive signals, various inhibitor compounds targeting LDH such as galloflavin (86), FX-11, gossypol (87, 88), NCI-006 (89), N-hydroxyindole-based inhibitors (90) and pyrazole based inhibitors (91)have been developed (**Figure 4**). However, recent demonstration of solid tumors downregulating the energetically expensive tissue specific functions such as glycolysis to allow uncontrolled growth despite a limited supply of ATP (92) should add a word of caution while choosing the drug targets. Moreover, targeting of lactate transporters with drugs such as cinnamate and AZD3965 can stop cancer cell proliferation (93, 94).

As explained before, lactate transporter inhibition diminishes cancer cells proliferation. On the other hand, inhibiting MCT protects immune cells from the risk of intracellular lactate accumulation. It has also been shown that knockout of MCT1 represses the function of immunosuppressive Treg cells and make the tumor environment conducive for antitumor immunity (61).

In addition to lactate, the accumulation of succinate is detected in the tumor microenvironment of some tumors. Tumor derived succinate impedes degranulation and cytokine (such as interferon-γ (IFN-γ)) secretion in both CD4 and CD8 T cells. In this situation T cells uptake more succinate (partly by MCT1) and accumulation of succinate into the cells inhibits succinyl coenzyme A synthetase activity and consequently, glucose flux through the tricarboxylic acid cycle is disturbed (95).

There are only a few studies that explored the combination of glycolysis metabolism targeted therapy with cancer immunotherapy. Genetic inhibition of glycolysis in tumor cells has been found to augment checkpoint blocker therapy (96). Accordingly, combination of 2-DG, BAY-876, and chloroquine and a glycolysis inhibitor nano-drug (D/B/CQ@ZIF-8@CS) has been shown to improve anti-CTLA-4 immunotherapy by reducing Treg metabolic fitness (97). Combination of Lonidamine, with anti-PD-1 therapy also has been shown to improve the therapeutic outcomes in glioblastoma mice model (98). The efficiency of anti-PD-1/PD-L1 therapy is also increased in pancreatic ductal adenocarcinoma cells (PDAC) which have deletion of PKM2 (99) implying strategies downregulating PKM2 in PDAC may synergize with ICI using anti-PD1/PD-L1.

Various therapeutic interventions may have differential effects on the ability of immune cells, particularly the effector T cells to migrate, infiltrate, and kill the tumor cells (96). Application of inhibitors of glycolysis or transport molecules such as MCT and GLUT may result in decreased lactate levels in the TME that would tend to alleviate the lactate mediated immune suppression and may also enhance immune cell infiltration (100). However, given the complexity of the TME and presence of tumor and immune cells in close proximation, it will be important to devise strategies for differential targeting of tumor and immune cells. One alternate can be ex-vivo treatment of immune cells for increasing the efficacy of adoptive cell therapy or CAR-T cell therapy. For example, culturing cytotoxic T cells or CAR-T cells under hypoxic conditions (101), or reducing culture conditions (102), or in the presence of appropriate inhibitors such as adenosine receptor inhibitors (103) seem to enhance the anti-tumor potential of these cells.

## Conclusion

Glycolysis is central to cell metabolism. However, its role goes far beyond the energy channel of cells. Various metabolites passing through the glycolytic pathway not only help in ATP synthesis but also in generation of reducing powers such as NAD. These reducing powers and other intermediates generated during the process of glycolysis are involved in cell signaling. Interestingly, these intermediates seem to be utilized differentially in tumor and immune cells. Thus, a thorough understanding of the regulatory factors that control a continued flow of energy and of various metabolites in cancer and immune cells will be helpful in devising differential targeting strategies. Such differential targeting strategies will be especially important in complex tumor microenvironment wherein tumor and immune cells reside in close contact, and it is difficult to target one cell type over the other. For example, lactate has been traditionally recognized as a tumor cell derived waste product and an oncometabolite that contributes to suppression of immune functions. However, it has become amply clear that in addition to being an immune suppressant, lactate also functions as an energy source in immune cells as at low concentrations, lactate can fuel the TCA cycle and can be used preferentially through TCA cycle. Hence, the role of metabolites of the glycolytic pathway, particularly the lactate is highly context dependent and may potentially be used for enhancement of the efficiency of cancer immunotherapy.

## Author contributions

SM and VV conceptualize and wrote the manuscript. Both authors confirm reading and approving the contents of the present manuscript. All authors contributed to the article and approved the submitted version.
